# Supplementary material for: Farmers’ willingness to pay for foot and mouth disease vaccine in different cattle production systems in Amhara region of Ethiopia
Source: PLoS One. 2020 Oct 2;15(10):e0239829. doi: 10.1371/journal.pone.0239829 (PMC7531826; doi:10.1371/journal.pone.0239829)
Supplement: S1 File — (DOCX) [file pone.0239829.s001.docx]

**S1 File. Willingness to Pay Questionnaire survey**

Part I. Willingness to pay question

1. Do you know the disease foot and mouth disease? if yes what are typical features of the disease (if the respondent mention one or more of the following clinical or epidemiological feature of the disease (s)he is considered as (s)he knows the disease
   - 1. Lameness ( foot lesion) and salivation (mouth lesion) in cattle and/ or small ruminants
     2. Foot lesion in cattle and/ or small ruminants that is contagious
     3. mouth lesion in cattle and/ or small ruminants that is contagious
     4. Lameness or mouth lesion, and blisters (sores)on teats of cows
2. The vaccine

The vaccine is a trivalent vaccine containing serotype O, A and SAT2. It will be given twice a year in the first year and then annually. The vaccine will be delivered by the government veterinary service coming into your village. The vaccine has an efficacy of 80% in protecting your cattle from FMD. To get this vaccine you need to pay XX ETB/ year. Don’t forget this cost is in addition to other household and business expenses that you have to meet.

- 1. Have you clearly understood the situation stated about the vaccine discussed above?

if no repeat the question until the respondent understood the scenario and if yes continue with questions below

- 1. Are you willing to pay the proposed price i.e. --- ETB/year for the vaccine explained above

1. yes b.no c. undetermined
   1. if yes to question 2.2 above, what if the vaccine price is ______ ETB) (50 % increase in the original price)
2. yes b.no c. undetermined
   1. if no to question 2.2 above what if the vaccine price is _________ETB/year (50 % decrease in the original price
3. yes b.no c. undetermined

**Part II. Socio demographic variables**

1. Respondents (household head) Name___________________
2. sex ____________
3. Age __________________
4. Education status___________________
5. Household size__________________
6. Main livelihood ____________
7. Contribution of livestock to livelihood a. main b. supplementary

**Economic variables**

1. No. of cattle owned ____________
2. Livestock (TLU) owned: #cattle, ___#sheep, ____#goat,_____ #donkey,____ #horse, ____#mule, ____#poultry____) .
3. No. cattle sold or income from the sale of cattle in the last one year __________
4. Quantity of milk sold or income from the sale of milk in the last one year _____
5. Quintal of crop harvested in the previous year ___________
6. Income from other agricultural activities (horticulture or forestry) in the last one year __________
7. Off farm income in the previous year _____________

**Husbandry and Disease control practices**

1. The breeds of cattle owned
   1. local breeds b. improved breeds c. mix of local and cross breed
2. Involvement in market oriented production like fatting and milk sale
   1. yes b.no
3. Frequency of use of modern veterinary service when faced with animal health problem
   1. always b. sometimes c. never
4. Traditional medicine and practices as source of animal health service
   1. main b. supplementary
5. Experience of using vaccine for livestock
   1. yes b.no

**Perception of Impact of FMD**

1. The frequency of FMD occurrence in your area
   1. Very low b. low c. moderate d. high e, very high
2. The impact of FMD on Milk production
3. Very low b. low c. moderate d. high e, very high
4. The impact of FMD on draught power
   1. Very low b. low c. moderate d. high e, very high
5. The impact of FMD in terms of Mortality
   1. Very low b. low c. moderate d. high e, very high
6. The impact of FMD on body condition
   1. Very low b. low c. moderate d. high e, very high
7. Cost of treatment of FMD ill animals
   1. Very low b. low c. moderate d. high e, very high

**Knowledge/ perception about livestock vaccines**

1. Vaccine are very useful to prevent disease
   1. Yes b. no c. don’t know
2. Vaccines do not cures affected animals
   1. Yes b. no c. don’t know
3. One vaccine is used only for one disease
   1. Yes b. no c. don’t know
4. Most Vaccines give protection only for limited time
   1. Yes b. no c. don’t know
