# Supplementary material for: Farmers’ willingness to pay for foot and mouth disease vaccine in different cattle production systems in Amhara region of Ethiopia
Source: PLoS One. 2020 Oct 2;15(10):e0239829. doi: 10.1371/journal.pone.0239829 (PMC7531826; doi:10.1371/journal.pone.0239829)
Supplement: Attachment — Submitted filename: Response to reviewers comments.docx [file pone.0239829.s003.docx]

**Responses to reviewers’ comments**

*Note: The line number refer to the Revised Manuscript with Track Changes*

Reviewer #1:

**Comment:** The study is an important one as it can lead to better control of FMD in Ethiopia. Though the appropriate data has been collected and most of the analyses done, the analysis mentions procedures like checking for multicollinearity but the details of how are missing. It is not clear how WTPs were constructed in the analysis. The resultant coefficients in the regression analysis are not interpreted at all, only the p values (significance).

Response:

- Detail of how multicollinearity was assessed for covariates in the model is provided in the revised version (L219-22)
- How the willingness to pay was derived from interval regression model was documented in L 212- 215 of the manuscript. It is explained in more detail below in the responses given for the specific comments on the issue.
- The interpretation of the model coefficients are provided in the revised version (L269-272)

Specifically:

L24: Replace ‘rarely’ with ‘sparingly’ and expensiveness with 'high cost'

Response: modified as suggested (L24)L34-37: There are too many variables in this sentence. One is unable to see what is being compared. Split the sentence to make clear what WTP is significantly higher than which with regard to systems, livelihoods, breeds

Response: The sentence split as suggested for easy reading (L34-37)

L40: Report in the affirmative rather than in the negative by turning the sentence around

Response: the sentence changed as suggested (L40-41)

L42: market-oriented system farmers

Response: modified as suggested (L 44)

L43-44: Be specific on which vaccine, which disease because your study was not on all vaccines, all diseases

**Response:** The vaccine knowledge was asked for livestock vaccine in general as farmers didn’t vaccinate against FMD and have no experience with FMD vaccine to give FMD vaccine specific information. This was stated in the methodology L177 and the appendix)

L56: Write UK in full; Scandinavia

Response: corrected as suggested (L 57)

L65: Do you mean endemic rather than epidemic?

Response: this was to refer to the epidemic nature of the diseases (FMD, PPR, LSD etc) which always occur in the form of outbreak instead of more or less constant number of cases throughout a year as endemic disease like mastitis or bovine paratuberculosis etc.

L67: Replace ‘vaccination’ with ‘application’ otherwise it reads like the vaccines are being vaccinated

Response: changed as suggested (L68)

L68: ‘negatively affect the effectiveness and raise the cost of vaccination’

Response. This was as compared to monovalent vaccines. When multiple strains are included in the vaccine, the host immune system exhaust and produce less immune response than when antigens are injected separately and hence reduce potency, and at the same time the cost increases because of inclusion of antigen load for each serotype. The sentences is modified to make this clear (L69-70)

L 75: Sheep and goat

Response: Corrected (L77)

L77: replace etc. with among others

Response: modified as suggested (L78)

L78: vaccine not vaccines

Response: corrected (L80)

L149: What does it mean ‘undetermined alternative’

Response: This is an option given to respondents when they could not decide ‘yes’ (agree) or ‘no’ (disagree) with proposed vaccine price. Including this option of “’undetermined or “no answer” is customary in the dichotomous bid design (see *reference 5* in the manuscript).

The survey should really be in reverse. You first get the perception and knowledge of the farmers on FMD as you need them as independent variables. Then you need to make all of them understand the disease and its control by the vaccine whose attributes they need to understand before bidding because as per your background information, these farmers may not even know the vaccine. Then you pose the bidding questions. Apparently before bidding you did not explain these aspects to the farmers which makes one wonder whether they knew what they were bidding for.

**Response**: The first question in the questionnaire was used to verify whether the respondent did know the disease (appendix). It was only if the farmer correctly described the disease that he/she continued for the rest of the survey (this was clearly stated in line 194). Next the questionnaire describes the attributes of the vaccine the farmers are bidding for such as about its effectiveness, how it delivered and its price etc. So the concern that “the bidding questions are posed before the farmers know what they were bidding for” is not practiced. While the reverse order could have been also possible as commented, our intention was first to estimate the amount of the willingness to pay and then identify what factors could affect this willingness.

L217: Change check to check; It is not clear how multicollinearity was checked
It is rare that a backward elimination of non-significant variables will result in a model with only significant variables.

Response: “checked’ corrected (L220)

Multicollinearity was checked using variance inflation factor (VIF) and VIF value above 10 was considered as indicator of presence of multicollinearity (see reference 22). This is made clear in the revised version (L219-222).

The significant variables reported in our manuscript are those which remain significant after back ward elimination. We started with the maximum model (all variables), run the model, identify the variable with least significant p-value and drop this variable and run again, we continued until only significant variables were left in the model.

L226: What does ‘inconsistent response’ mean?

Response. If the answer for the second question cannot logically go with the answer for first question it is said inconsistent. For example. If the respondent answered he/she has no cattle for the first question, he/she should not answer the question about the breed of the cattle asked in the next question.

L227: Who gave the ‘undetermined response’ for WTP?

Response: as choices were “yes”, “no” and “undermined” (see the annexed survey) few respondents answered “undetermined’

L230: The ‘majority’ here is only about half. How were the results of the other half? Explain from the table without repetition

Response: by majority we mean 50 +1. In this question the main interest whether livestock is main source of income or not and whether willingness to pay is strong when the main source of income is livestock. So the other sources of income (crop farming, trade, employment etc.) were categorized simply as others in the analysis. We indicated this in Table 2 of the current version. But we are afraid it will be irrelevant to list the proportion of farmers with each of these income sources

Table 2: In the column, align contents conventionally; text aligned to the left and figures to the right. What is income from cattle and milk sale? Annual, monthly or what?

Response: the table formatted as suggested. The income from milk and cattle sales were asked for the preceding one year. This is indicated in the table as suggested. (Table 2)

L255-260: It is not clear how the mean WTPs were calculated

Response: Here the willingness to pay was determined form a constant only (null) interval regression model. The constant of this model is the mean willingness to pay just as the constant term of a linear regression model is the mean value of the dependent variable except that the constant in interval regression is determined using maximum likelihood estimation instead of simple average of observed values of the dependent variable as in linear regression. This estimate of willingness to pay is without the use of the knowledge of socio-demographic and husbandry characteristics of the respondents. Later in Table 6 the willingness to pay was estimated taking into account socio-demographic and husbandry characteristics of the respondents using the best model built from the potential predictors (see the response for a similar comment below). The detail of the modelling can be found in *reference 21* of the manuscript.

L262: The p should be >0.05 if no significant association

Response: The place and sign of the p value is changed for more clarity (L267)

Table 4: Just indicate the reference category within the table by writing ref in the model coefficients column for the respective reference categories instead of having a footnote which is not clear

Response: modified as suggested (Table 4 and 5)

L261-265 and 272-278: Give the interpretation of the model coefficients, not just of the p value (significance). Considering the regression equation that arises from the results would help

Response: example of the interpretation of model coefficients has been given (L270-273). Interpreting all the coefficients would be monotonous as they can be easily seen in the table.

L272-276: Remove unnecessary capitalization of words

Response; The unnecessary capitalizations are corrected (L281-282)

Table 6: It is not clear how the WTP estimates were derived

As explained above for a similar comment. Here willingness to pay estimates were derived form the best interval regression models built (table 4 for all respondents and table 5 for MCL and MO respondents separately). The best models were used to predict the willingness to pay for each respondent. From these individual estimates mean and median willingness to pay were calculated. The estimation using the mode has been described in L 213-216 and detail can be found in *reference 21*.

L300: citation/reference?

Response: Theory for demand is changed into law of demand and reference has been given (L309)

L311: What is ‘parametrized’?

Response: Fitting the model with data obtained from double bound dichotomous questioner survey

L321-322: Correct the sentence

Response: the sentence is corrected (L330)

L328: What is ‘quality vaccine’ – you did not explain the vaccine to them

Response: the quality of vaccine was described to the farmers (in the appendix, question 2). Its efficacy was stated as 80%. According to OIE a standard potency FMD vaccine with protection percentage of 75% and above is considered as quality FMD vaccine. This has been also described L162-164

L330: What is CLM?

Response: sorry it was to mean MCL; corrected (L328)

L332: Change ‘tend’ to ‘tends’(L341)

Response: corrected

L348: Avoid an abbreviation at the beginning of a sentence

Response: written in full as suggested (L358)

L362-365: Sentence is too long and needs rephrasing for clarity

Response: the sentence is split into two to make it clear (L373-375)

L374: Correct the word knowledge

Response: corrected (L385)

In the conclusion, WTP alone is not sufficient to make the conclusion that vaccine price can be covered by farmers fully without your knowledge on their ‘ability to pay’. You need to look at their profits or how they perceive profitability from their enterprises. As is, it is possible that WTP may represent valuation of vaccination rather than actual desire to pay the amounts

Response: The willingness to pay question was asked whether they are willing to pay for the vaccine at the stated prices. So If they answer yes, it is assumed that they think that it will be worthwhile (profitable) investment to pay that amount (see L 315-317 where farmers implied this rationale) and will pay if they are asked that amount for the prevention of the disease.

L441: correct 20.20

Response: corrected (L452)

**Reviewer #2: Reviewer comments**:

Excellent study design, data analysis, and overall manuscript. A few suggestions below. Suggest one more read through by a strong English speaker to remove typos. Identified some below, but there are probably more, and they distract from the quality of the paper.

Response: Serious proofreading has been done to minimize the grammar and typographical errors

Line 35-36: should read “whose main livelihood is”
36 “livestock than those whose main livelihood is other than livestock,” – Rewrite, confusing

Response: the sentence is broken down for more clarity (Line 35-37).

65 – typo – should read peste

Response: corrected (Line 66)

63 onwards – May be worthwhile to mention the role of diagnostic testing in FMD vaccination as an additional challenge – alluded to but not directly mentioned

Response: Yes, most of the time vaccination complicates diagnostic testing when the vaccines are not DIVA vaccines. But for FMD there are tests which can differentiate vaccinated and non vaccinated animals such as 3ABC ELISA which are based nonstructural proteins of the virus. So relatively this is not a major problem for FMD as compared to other important disease such as PPR where such tests or DIVA vaccines are yet not developed.

78 – should read “vaccine is…”

Response: corrected (Line 80)

120 – contingent valuation is also appropriate in this situation because many people are not vaccinating.

Response : Yes, this is addressed in Line 124-3 on ‘poor adoption”

180 – should read “asked for”

Response: corrected as suggested (Line 184)

198 – should read farmers’

Response: corrected (Line 201)

199 – should read double-bounded (Line 201)

Response: corrected

347 – should read “is the main”

Response: corrected (Line356)

370 – should read “are willing to pay more”

Response: revised as suggested (Line 380)

373 – should read “pay more”

Response: revised as suggested (Line 384)

381 – should read farmers’

Response. Corrected (Line 392)

Response: corrected

385 – Should read farmers

Response: corrected (Line 396)

Gender – noticed the percentage of women respondents is very low. Do you think this is representative of the study population? Seems possible the selection of participants may bias against women if they are more likely to be at home. May be worthwhile to think more about this in the discussion.

Response: Clarification about this is added under sampling. The respondents were the head of households (Line 194) and in that setting the heads of the households are mainly males.
