# Supplementary material for: Farmers’ willingness to pay for foot and mouth disease vaccine in different cattle production systems in Amhara region of Ethiopia
Source: PLoS One. 2020 Oct 2;15(10):e0239829. doi: 10.1371/journal.pone.0239829 (PMC7531826; doi:10.1371/journal.pone.0239829)
Supplement: Attachment — Submitted filename: Response to the reveiwers comments.docx [file pone.0239829.s004.docx]

**Response to the reviewer comments**

***NB: the line numbers refer to the revised version of the Manuscript with track changes***

23 vaccines

**Response**: corrected (L23)

38 Remove “was also seen”

**Response**: modified as suggested (L38)

39-43 “perception that farmers would be reluctant to pay for FMD vaccine 41 is unprovable” – Be careful about how you word this. You have measured a distribution of WTP and some farmers may still have reluctance to pay. Review scientific method and do not talk about results suggesting something is unprovable.

**Response:** the sentence modified to address the concern (L39-41).

“if official FMD vaccination is planned in the country, the vaccine cost can be covered” – Do not confuse WTP with what people will actually pay in a real life scenario. Higher WTP means respondents value the vaccine more but it does not necessarily mean “WTP scenario money” is exactly the same as real life money. Reword to say something like “market-oriented farmers with higher willingness to pay may be more likely to pay full cost if official FMD vaccination is planned in the country than mixed crop livestock farmers”

**Response**: the interpretation of the results are revised to address the concern of overstating the results and the conclusions are restated accordingly (L39-44, 391-393). Moreover, in the discussion section, it has been discussed that contingent evaluation tends to overestimate the WTP as compared to actual market behavior and caution should be taken for practical application the results (L332-35).

51 “the most important disease of livestock worldwide” – subjective statement. Reword.

**Response**: reworded as “ARGUABLY the most important disease of livestock worldwide” (L52)

56 Scandanavia

**Response**: corrected (L57)

57 the case

**Response**: corrected (L58)

63 onwards – I was not referring to DIVA vaccines but that without diagnostic testing, FMD vaccines can be poorly matched to the circulating serotype and therefore less effective. Is this applicable in Ethiopia? Address this as a constraint if so. <https://www.ncbi.nlm.nih.gov/pmc/articles/PMC7067263/>

Response: Yes. There are multiple serotypes of FMD virus and vaccine matching is an issue in Ethiopia as well. This problem with FMD vaccine has been mentioned in this revision (L68-70).

66 replace cheap with affordable

**Response**: replaced as suggested (L67)

77 pox, among others,

**Response**: corrected as suggested (L79)

138 – knew – USE SPELL CHECK!! (Spelling checked as suggested (L 145))

– It is reasonable to exclude farmers who are not familiar with FMD, but this will bias your WTP estimates up. You should then be using even more caution about the statements in lines 39-43. You have systematically removed farmers likely to be reluctant to pay then claimed farmers will cover costs. Be more conservative when wording conclusions.

**Response:** as stated in the previous comment, the conclusions have been revised to address the concern with the over optimistic interpretation of the results (L39-44, 391-393).

296 Yes yes can also mean you truncated the distribution of the higher end of the WTP distribution curve.

**Response**: correct. ‘yes’ ‘yes’ mean the upper bound for these respondents is higher than stated bids and shows the farmers are enthusiastic for the vaccine.

302 – This suggests vaccines are valued but it does not necessarily mean farmers will pay the amount stated in a hypothetical scenario. Do not misinterpret as such.

**Response:** the statement here just compared the stated mean willingness to pay to the actual price and does not necessary imply that they will pay that amount.

364 – List threshold used when testing for multicollinearity

**Response:** already mentioned in the material and method part i.e. VIF equal or greater than 10 was considered as indicator of multicollinearity (L225-26)

382 – See previous comments. Overstating your results again.

R**esponse**: the conclusion revised to avoid the overoptimistic interpretation (L391-393)

Supporting information – misspelled supplementary

**Response:** modified as supporting information (S1)
Overall: Do not overstate results – be more careful in how the conclusions are worded. Please check English again and use spell check. Include dataset of WTP bids and relevant variables from the survey referenced in the text.

**Response:** the overoptimistic interpretation of results are revised and the conclusion are restated accordingly (L39-44, 391-393). Spell check has been done to avoid language errors and the data set for the WTP are included as supporting information (see S2)
